# Supplementary figures and images for: The clinical value of progestin-primed ovarian stimulation protocol for women with diminished ovarian reserve undergoing IVF/ICSI: a systematic review and meta-analysis
Source: Front Endocrinol (Lausanne). 2023 Aug 21;14:1232935. doi: 10.3389/fendo.2023.1232935 (PMC10476097; doi:10.3389/fendo.2023.1232935)

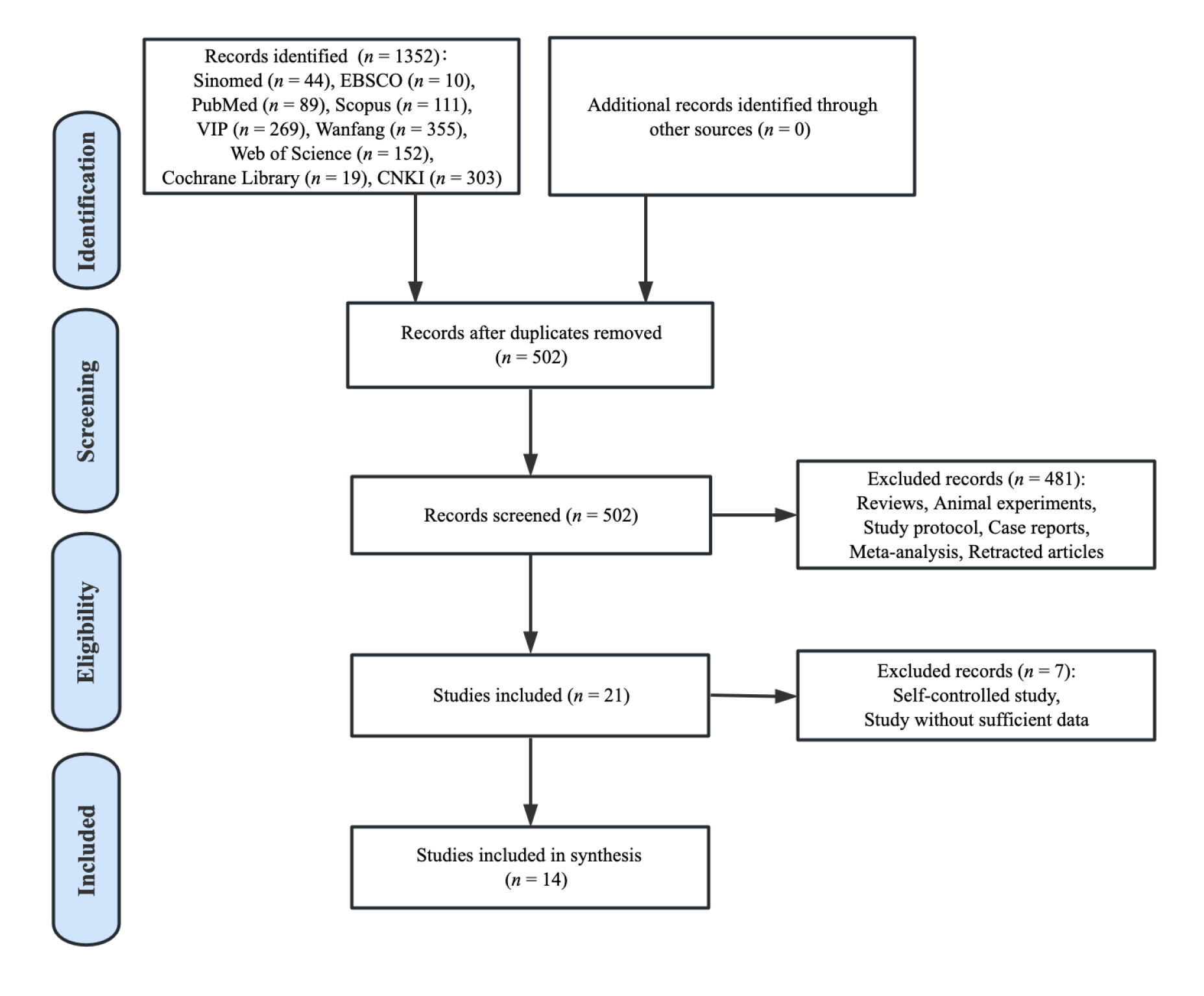

Supplement: Supplementary file 1 [file Image_1.tif]
