# Supplementary material for: The clinical value of progestin-primed ovarian stimulation protocol for women with diminished ovarian reserve undergoing IVF/ICSI: a systematic review and meta-analysis
Source: Front Endocrinol (Lausanne). 2023 Aug 21;14:1232935. doi: 10.3389/fendo.2023.1232935 (PMC10476097; doi:10.3389/fendo.2023.1232935)
Supplement: Supplementary file 2 [file Table_1.doc]

**The clinical value of progestin-primed ovarian stimulation protocol for women with diminished ovarian reserve undergoing IVF/ICSI: a systematic review and meta-analysis**

Guangyao Lin^1^, Xiufang Zhong^2^, Shengnan Li^1^, Xiyu Liu^1^, Lianwei Xu^1*^

1. Department of Gynecology, Longhua Hospital, Affiliated to Shanghai University of Traditional Chinese Medicine, Shanghai 200032, China.
2. Department of Reproductive Center, Shuguang Hospital Affiliated to Shanghai University of Traditional Chinese Medicine, Shanghai 201203, China.

^*^Corresponding author. E-mail addresses: xu_lianwei2800@shutcm.edu.cn;

**Abstract**

**Background:** To determine whether progestin-primed ovarian stimulation (PPOS) is more effective for women with diminished ovarian reserve (DOR) than clomiphene citrate (CC) /letrozole (LE) plus gonadotropin in IVF or ICSI treatment.

**Methods:** Nine databases were searched until May 24th, 2023, to identify relevant studies. Forest plots were used to present the result of this meta-analysis. Begg’s and Egger’s tests were applied to estimate publication bias. Subgroup and sensitivity analysis were performed to check the potential sources of heterogeneity and verify the robustness of the pooled results, respectively.

**Results:** A total of 14 studies with 4182 participants were included for meta-analysis. There was evidence of a statistically notable increase in clinical pregnancy rate (OR = 1.39, 95%CI [1.01, 1.91], *p* = 0.05), optimal embryos rate (OR = 1.50, 95%CI [1.20, 1.88], *p* = 0.0004), and cumulative pregnancy rate (OR = 1.73, 95%CI [1.14, 2.60], *p* = 0.009), the duration and the amount of gonadotropin required (MD = 1.56, 95%CI [0.47, 2.66], *p* = 0.005; SMD = 1.51, 95%CI [0.90, 2.12], *p* < 0.00001), along with decrease cycle cancellation rate (OR = 0.78, 95%CI [0.64, 0.95], *p* = 0.02), luteinizing hormone (LH) level on the day of hCG (SMD = -0.81, 95%CI [-1.10, -0.53], *p* < 0.00001), and premature LH surge rate (OR = 0.10, 95%CI [0.07, 0.15], *p* < 0.00001) when PPOS was used. No evidence for publication bias within result was revealed.

**Conclusions:** Based on evidence-based results, PPOS protocol seems to improve IVF/ICSI outcomes for women with DOR. More research with larger sample sizes and rigorous designs are required to clarify the value of PPOS among women diagnosed with DOR.

**Keywords:** diminished ovarian reserve, progestin-primed ovarian stimulation, clomiphene, letrozole, in vitro fertilization, intracytoplasmic sperm injection

**1 Introduction**

Infertility is a severe health problem and affects 9% reproductive-aged women globally (1). The incidence of infertility has grown substantially, and it is estimated to impact 186 million people in the 21st century (2). One of the primary causes of infertility is diminished ovarian reserve (DOR) (3). A recent statistic based on 181,536 assisted reproductive technology (ART) cycles demonstrated that the overall prevalence of DOR is estimated to be 19 to 26% in the US (4). Furthermore, DOR, characterised as decreased oocyte quality and quantity, is significantly associated with poor reproductive outcomes, which is still a serious clinical challenge for ART treatment (5,6). Numerous studies indicated indicate that infertile women with DOR experienced higher miscarriage rates, lower opportunity chance of possessing at least one euploid blastocyst, increased risk of cycle cancellation, and poor ovarian response in in-vitro fertilization (IVF) (7-10). Therefore, it is imperative to explore appropriate ovarian stimulation protocols to improve the outcomes for women with DOR undergoing ART.

Currently, no guideline or consensus recommends an applicable ovarian stimulation protocol for women with DOR. In clinical practice, pituitary suppression and gonadotropins are wildly widely employed to prevent premature luteinizing hormone (LH) surge and ovulation in the course of IVF or intracytoplasmic sperm injection (ICSI) cycles. Despite their overall effectiveness, ovarian hyperstimulatory syndrome, reduced live birth rate, worse oocyte quality, and higher medication costs often occur in high-dose gonadotropins stimulationDespite their overall effectiveness, high-dose gonadotropins stimulation is often accompanied with ovarian hyperstimulation syndrome, reduced live birth rate, worse oocyte quality, and higher medication costs (11-13). In addition, gonadotropin-releasing hormone (GnRH) applied in pituitary suppression has had been proven that 0.34% to 8.0% failure fail to manage premature LH surge, the significant risk factor associated with DOR(14). Moreover, GnRH antagonist protocol in IVF cycles could increase uterine natural killer cells and tumour necrosis factor α, which negatively affects endometrial receptivity (15). Clomiphene citrate (CC) and letrozole (LE) are often administered in IVF treatment for ovarian stimulation. A retrospective cohort study has revealed that the live birth rate was significantly lower in CC cycles compared to that with natural cycles (*p* = 0.01), whose underlying mechanism might be that CC influenced uterine receptivity by reducing endometrial thickness through antioestrogenic effects (16). Besides, the use of LE during inducing ovulation has been reported to be correlated with a notable risk of elevating progesterone levels, which has an adverse effect on the pregnancy rate (17). Hence, over the past few years, an alternative approach known as progestin-primed ovarian stimulation (PPOS) in controlling the LH surge has attracted lots of clinicians. Observational studies have demonstrated that PPOS generated a similar formation of euploid blastocysts per oocyte, live birth rate, cumulative ongoing pregnancy, and metaphase II oocytes (MII), along with 2 pronuclear fertilized oocytes (2PN) with GnRH antagonist protocol (18-20). Simultaneously, a randomised parallel controlled trial involving 300 infertile women revealed that PPOS resulted in similar outcomes, including clinical pregnancy rate, the number of oocytes retrieved, and live birth rate, when compared with human menopausal gonadotropin (HMG) Simultaneously, a retrospective cohort study involving 3556 infants revealed that PPOS resulted in similar neonatal outcomes, including the early neonatal death, preterm birth, rates of low birthweight, and large/small-for-gestational age, when compared with GnRH agonist short protocol. (21).

However, several clinical studies investigating the value of PPOS protocol for women with DOR undergoing IVF or ICSI produced conflicting results. For example, Liu et al. (22) included 108 cases and showed that PPOS protocol during IVF has the same clinical pregnancy rate, optimal embryos rate, and cycle cancellation rate compared to clomiphene citrate (CC) plus gonadotropin stimulation, which is contrary to Zhao’s study (23). Meanwhile, Fu et al. (24) demonstrated that the number of oocytes retrieved, optimal embryos rate, and cycle cancellation rate were not improved with PPOS protocol compared with CC plus LE stimulation. Still, Zhang’s study (25) confirmed that the PPOS protocol group achieved more oocytes retrieved, optimal embryos rate, and lower cycle cancellation rate than CC plus LE group. The divergent conclusions above may be inaccurately and insufficiently estimated because of the limited sample size from single clinical research. Therefore, we performed this meta-analysis to summarize the existing evidence quantitatively and inform clinical practice. The study’s specific concern was as follows: Does PPOS improve the outcomes for women with DOR undergoing IVF or ICSI compared to CC/LE plus gonadotropin stimulation?

**2 Materials and methods**

This study (PROSPERO registration No. CRD42023430202) was conducted following the preferred reporting program of the systematic review and meta-analysis (PRISMA) (26).

- 1. **Search strategy**

We thoroughly searched nine databases, including English-language databases Cochrane Library, Sinomed, EBSCO, Web of Science, Scopus, PubMed, and Chinese-language databases Wanfang, VIP Information, and China National Knowledge Infrastructure (CNKI) from inception up to May 24th, 2023. The search strategy was composed of two components: clinical condition (decreased ovarian reserve, declined ovarian reserve, diminished ovarian reserve), intervention (assisted reproduction technology, ICSI , IVF, mild stimulation, microstimulation, progestin primed ovarian stimulation, clomiphene plus gonadotropin, letrozole plus gonadotropin). We included use different combinations of the following search terms: “decreased ovarian reserve”, “declined ovarian reserve”, “diminished ovarian reserve”, and “assisted reproduction technology”, “ICSI”, “IVF”, “mild stimulation”, “microstimulation”, “progestin primed ovarian stimulation”, “clomiphene plus gonadotropin”, “letrozole plus gonadotropin”. The first two authors (G.Y.L. and X.F.Z) independently screened the articles through titles, abstracts, or full text to identify the eligibility of the studies. In addition, we carefully screened the references from retrieved studies to obtain more relevant research as much as possible.

- 1. **Inclusion and exclusion criteria**

The studies met the following criteria would be included: (1) studies of patients who were diagnosed with DOR (AFC<5~7 or FSH ≥ 10IU/L or AMH < 1.1ng/mL) (27,28); (2) all patients received ART treatment, including IVF and ICSI; (3) studies divided patients into two groups in term of ovarian stimulation protocols (PPOS versus CC/LE plus gonadotropin) regardless of the types of progestin; (4) studies reported the outcomes of PPOS treatment with sufficient data studies provided the diagnostic criteria for DOR and basal characteristics (i.e,. age, duration of infertility) of patients with sufficient data; (5) if the data were part of the same project, the article with the largest sample size was included; (6) type of studies were randomised controlled trials, observational studies, and cross sectional studies; (7) there were no ethnic and geographical restrictions.

During the selection process, the excluded studies satisfied the following criteria The exclusion criteria included: (1) researches were self-controlled study; (2) patients with polycystic ovary syndrome, abnormal endometrium, intrauterine adhesion, uterine malformation, reproductive tumors, and chromosomal abnormalities; (3) meta-analysis, study protocol, duplicate publications, reviews, animal experiments, and conference papers; (4) studies did not publish in Chinese or English.

- 1. **Data extraction and Quality assessment**

A standardized form was adopted by G.Y.L. and X.F.Z. to perform data extraction independently. The following data were retrieved: study population characteristics (i.e., age, duration of infertility, body mass index), details of the treatments (i.e., type of gonadotropin, intervention of ovarian stimulation protocol), and outcomes in each group. The primary outcomes were cycle cancellation rate, clinical pregnancy rate, the number of oocytes retrieved, and premature LH surge rate. The secondary outcomes were optimal embryos rate, fertilization rate, live birth rate, cleavage rate, embryo implantation rate, estradiol (E_2_) and LH on the day of hCG, duration of gonadotropin used, total dose of gonadotropin, cumulative pregnancy rate, and early miscarriage rate. Meanwhile, the Newcastle-Ottawa scale (NOS) was utilized by two independent reviewers (G.Y.L and X.F.Z) to evaluate the quality of the included articles. Studies were considered of high quality if studies with scores of ≥ 6 (29). Any discrepancies were determined by talking discussing with the corresponding author (L.W.X).

- 1. **Statistical analysis**

Analysis of the data was conducted with Review Manager 5.3 and Stata 15.1 software. Documents management was performed with EndNote 20.2 software. The continuous variables (for example, the number of oocytes retrieved) were presented with a standardized mean difference (SMD) or mean difference (MD) with 95% confidence intervals (CIs). For dichotomous variables (for example, cycle cancellation rate) , odds ratios (OR) with 95% Cls were shown. The heterogeneity in the meta-analysis was estimated by utilizing the *I^2^* statistic. An *I^2^* ≥ 50% was considered massive heterogeneity; then, the random-effects model was used. Otherwise, the fixed-effects model was adopted. Besides, the potential sources of heterogeneity were checked by subgroup analysis. *p* ≤ 0.05 was deemed statistically significant. Furthermore, Begg’s and Egger’s tests were applied by evaluating the P value to explore publication bias if at least ten studies were involved. When the *p* value was > 0.05, it is considered that there was no publication bias existing. If at least five studies were included, a sensitivity analysis was adopted by excluding individual articles to appraise the robustness of the pooled results.

**3 Results**

**3.1 Included articles**

The flow chart exhibited in the PRISMA figure (**Figure 1**) shows the selection of records included. The search strategy identified 1352 articles via database searching. After removing duplicates, 850 papers were excluded. Of the 502 studies identified, 481 studies were removed because they met the basic exclusion criteria when going through the titles and abstracts. After the full-text screening, seven relevant articles were further excluded as they were self-controlled studies or without sufficient data. Ultimately, a total of 14 studies were included in the analysis of this review.

**
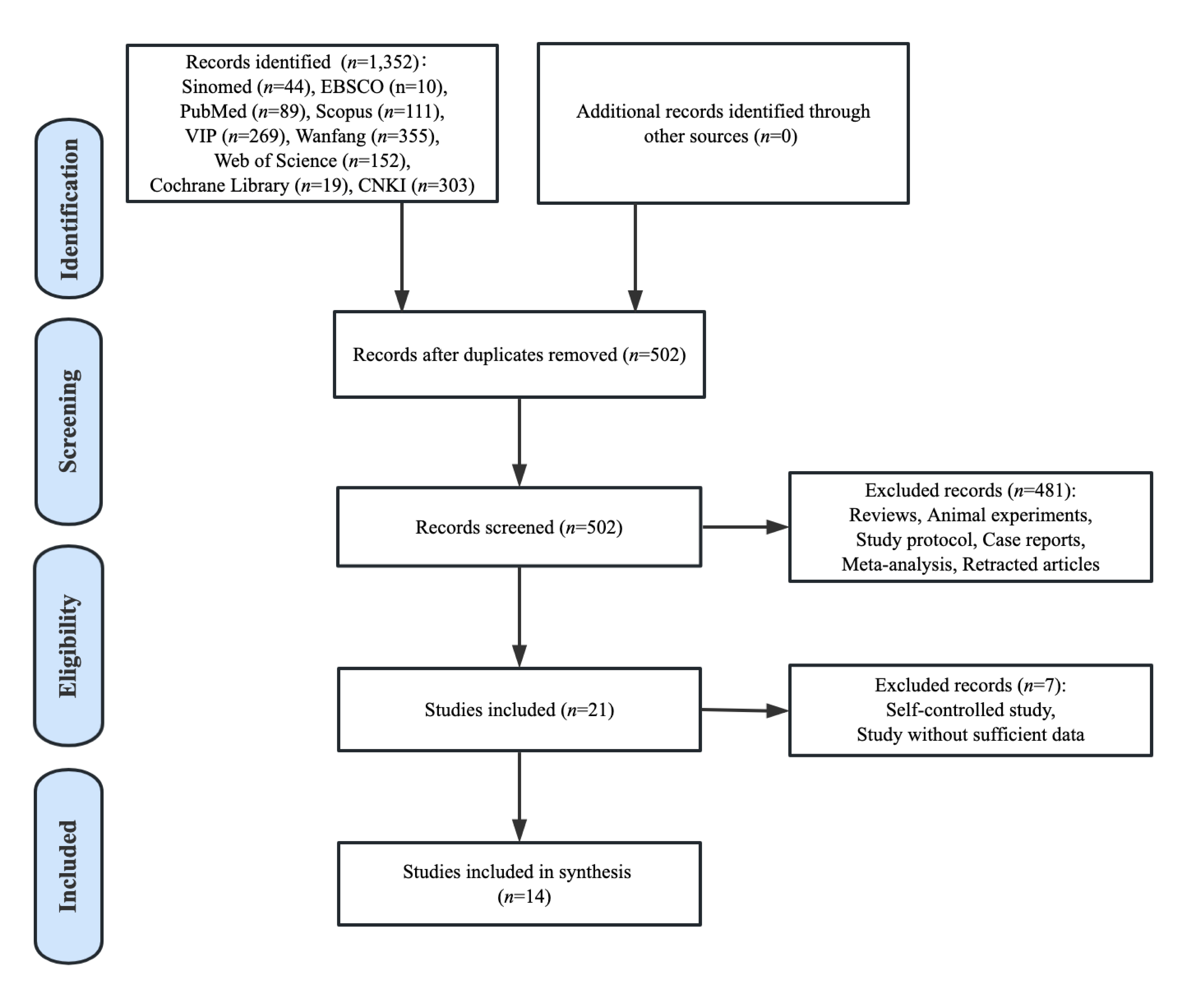
**

**FIGURE 1** Paper selection flowchart.

**3.2 Study Characteristics**

Table 1 shows the main characteristics of all included studies. We included 14 studies involving 4182 women with DOR undergoing IVF/ICSI treatment, with the sample sizes ranging from 65 to 972 with the sample sizes in each trial varying from 65 to 972 and the published year running from 2016 to 2023. All the patients included were from China and were divided into trial group treated with PPOS and control group treated with CC/LE plus gonadotropin. Each group The trial group and the control group comprised 2282 and 1900 cases, respectively. Four studies (23,30-32) applied the median and 25th - 75th percentiles for continuous variables with skewed distributions. In the control group, nine studies reported patients treated with CC, two studies treated with CC plus LE, one study received CC or CC plus LE, and the remaining two studies were CC or LE. Further, the characteristics of sex hormones level and AFC were also presented in supplementary table 1.

TABLE 1 Study characteristics.

| Study | Year | Sample size (n) | Age (years) | | Duration of infertility (years) | | BMI (Kg/m^2^) | | Gn | Intervention | | Outcomes | NOS |
| --- | --- | --- | --- | --- | --- | --- | --- | --- | --- | --- | --- | --- | --- |
|  |  | T/C | T | C | T | C | T | C |  | T | C |  |  |
| Tu (30) | 2022 | 600/139 | 39.45±2.99 | 39.98±3.34 | 3(6–2) | 3(5–1) | 22.03±2.62 | 22.29±3.00 | HMG | MPA | CC/LE | ①②⑥⑧⑫⑬⑮ | 8 |
| Fan (33) | 2021 | 486/486 | 40.14±3.72 | 40.25±3.43 | 4.31±2.96 | 4.35±2.79 | 21.53±2.66 | 21.38±2.71 | HMG | MPA/MPA+EE | CC | ①④⑤⑦⑪⑫⑬ | 8 |
| Yang (34) | 2022 | 47/247 | 39.00±7.00 | 40±6.00 | 3.00±4.00 | 3.00±4.00 | 23.20±3.80 | 22.90±4.40 | HMG | MPA | CC | ②④⑨⑩ | 7 |
| Fu (24) | 2017 | 87/96 | 40.10±4.50 | 39.90±4.80 | 6.80±4.10 | 6.30±3.90 | 22.54±4.07 | 22.00±2.84 | HMG | DYG+LE | CC+LE | ①③④⑤⑦⑨⑩⑪⑫ | 6 |
| Zeng (35) | 2020 | 103/123 | 38.19±4.96 | 37.98±4.85 | 5.56±4.9 | 4.88±4.12 | 22.85±2.57 | 22.34±2.76 | FSH/HMG | MPA | CC | ②④⑨⑩⑪⑫ | 7 |
| Zhao (A) (23) | 2023 | 61/65 | 35.00, 41.00 | 35.00,42.00 | 1.50,5.50 | 1.00, 8.50 | 23.57±2.78 | 23.68±2.20 | HMG | DYG | CC | ①③⑥⑭ | 7 |
| Zhao (B) (31) | 2023 | 41/45 | 41.0(38.0,43.0) | 41.0(38.0,42.0) | 2.0(1.0, 4.0) | 2.0(1.0,4.5) | 21.3(19.35,23.56) | 21.72(20.07,25.25) | Gn | PC | CC/LE | ①②③⑥⑦⑧⑬⑮ | 7 |
| Zhang (25) | 2016 | 94/70 | 37.5±5.7 | 36.8±5.3 | 5.7±4.1 | 5.0±3.8 | NA | NA | Gn | MPA | CC+LE | ①③④⑤⑦⑨⑩⑪⑫ | 7 |
| Zheng (36) | 2020 | 59/57 | 38.41±3.51 | 38.67±3.34 | 4.44±3.02 | 5.62±2.15 | 22.17±3.99 | 21.78±4.09 | FSH | PC | CC | ①④⑩⑪⑫⑭⑮ | 8 |
| Wang (37) | 2020 | 28/37 | 38.8±5.3 | 38.2±6.1 | 6.9±1.7 | 6.8±1.6 | NA | NA | FSH | MPA | CC/CC+LE | ④⑨⑩⑭ | 7 |
| Liu (22) | 2020 | 52/56 | 41.13±3.01 | 41.16±2.63 | 4.02±2.43 | 4.52±3.03 | 22.34 ±2.03 | 22.77±1.97 | Gn | MPA | CC | ①②③④⑧⑨⑩⑪⑫⑮ | 6 |
| Xu (38) | 2021 | 310/155 | 41.56±3.70 | 42.32±3.78 | 5.53±4.91 | 6.27±5.61 | 23.28±2.84 | 23.56±2.87 | FSH | MPA | CC | ②③④⑤⑦⑧⑨⑩⑪⑫⑮ | 7 |
| Yu(A) (39) | 2017 | 209/222 | 39.87±5.41 | 40.47±5.71 | 5(2,8) | 4(2,8) | 23.57±3.61 | 23.78±3.42 | FSH/HMG | MPA | CC | ①②⑮ | 8 |
| Yu(B) (32) | 2019 | 105/102 | <38 | <38 | 4.42±0.38 | 3.63±0.25 | 22.4±0.34 | 22.5±0.31 | HMG | MPA | CC | ①④⑦⑨⑩⑪⑫⑭ | 7 |

Abbreviations: T, trial group; C, control group; BMI, body mass index; Gn, gonadotropin; NA, not available; HMG, human menopausal gonadotropin; MPA, medroxyprogesterone acetate; CC, clomiphene citrate; LE, letrozole; EE, ethinyl estradiol; DYG, dydrogesterone; PC, progesterone capsules; FSH, follicle stimulation hormone; ① Cycle cancellation rate; ② Clinical pregnancy rate; ③ Optimal embryos rate; ④ Number of oocytes retrieved; ⑤ Fertilization rate; ⑥ Live birth rate; ⑦ Cleavage rate; ⑧ Embryo implantation rate; ⑨ E_2_ on the day of hCG; ⑩ LH on the day of hCG; ⑪ Duration of gonadotropin used; ⑫ Total dose of gonadotropin; ⑬ Premature LH surge rate; ⑭ Cumulative pregnancy rate; ⑮ Early miscarriage rate.

**3.3 Quality assessment**

All research was retrospective cohort studies, and the risk of bias and quality assessment were estimated in accordance with the NOS. Four of them were rated eight scores. Eight studies obtained seven scores, and two were evaluated as six scores. Although all studies assessed were of high quality, common reasons attributed to score low on study quality assessment was lack of sufficient detail outcome assessment procedures. Table 1 presents the NOS score of each study included.

**3.4 Outcome measurements**

**3.4.1 The primary outcomes**

Ten studies investigated the association between PPOS and cycle cancellation rate. Among the DOR women, PPOS demonstrated a favourable result for cycle cancellation rate with the pooled OR being 0.78, (95% CI: 0.64, 0.95), *I^2^*= 41%, *p* = 0.02 when compared with those women treated with CC/LE plus gonadotropin stimulation. Seven studies reported clinical pregnancy rate in patients with PPOS intervention. The result showed that the pooled OR was 1.39, (95% CI: 1.01, 1.91), *I^2^*= 0%, *p* = 0.05, revealing a higher rate of clinical pregnancy rate for women treated with PPOS compared with the control group. Furthermore, ten studies estimated the association of PPOS with the number of oocytes retrieved; after excluding Yu’s study (32) by sensitivity analysis, the heterogeneity decreased from 99% to 78%; therefore, the pooled MD was 0.30 (95% CI: -0.03, 0.62), *I^2^*= 78%, *p* = 0.08, suggesting that PPOS nearly yielded the same the number of oocytes retrieved with the control group. In addition, four studies with 1,905 cases reported premature LH surge rate. There was evidence of a notable decrease in premature LH surge rate when PPOS was used (OR 0.10, [95% CI: 0.07, 0.15], *I^2^*= 0%, *p* < 0.00001) (**Table 2**, Supplement Figure 1 **Figure 2**).





**FIGURE 2** Forest plot of studies evaluating cycle cancellation rate (A); clinical pregnancy rate (B); number of oocytes retrieved (C); premature LH surge rate (D).

**3.4.2 The secondary outcomes**

The optimal embryos rate was reported by six studies. After removing Zhao’s study (31) by sensitivity analysis, the heterogeneity declined from 58% to 44%; thus, there was evidence of a substantial increase in optimal embryos rate with PPOS compared to CC/LE plus gonadotropin (OR 1.50, [95% CI: 1.20, 1.88], *I^2^*= 44%, *p* = 0.0004). However, it was noteworthy that there was no evidence of a statistically striking difference in fertilization rate, live birth rate, cleavage rate, embryo implantation rate, E_2_ on the day of hCG, and early miscarriage rate between the groups (*p* > 0.05). Furthermore, nine studies investigated the association between PPOS with LH on the day of hCG, after removing Yu’s study (32) through sensitivity analysis, the heterogeneity reduced from 98% to 84%; among the women with DOR, PPOS was also shown to be an advantageous outcome (SMD -0.81, [95% CI: -1.10, -0.53], *I^2^*= 84%, *p* < 0.00001) (**Figure 3**).

In addition, the duration of gonadotropin used was measured in eight trials, and the total dose of gonadotropin was recorded in nine studies. The pooled results indicated that the PPOS protocol may statistically increase the duration of gonadotropin used (MD 1.56, [95% CI: 0.47, 2.66], *I^2^*= 97%, *p* = 0.005), and total dose of gonadotropin required (SMD 1.51, [95% CI: 0.90, 2.12], *I^2^*= 98%, *p* < 0.00001). Although sensitivity analysis was utilized, no individual research impacted the pooled results. Simultaneously, subgroup analysis based on different types of gonadotropin (HMG vs. FSH) explored the potential heterogeneity, but the heterogeneity did not modify (**Supplementary Figure 1**). What's more, four studies reported cumulative pregnancy rate, and the pooled result showed that PPOS was more superior to CC/LE plus gonadotropin in increasing cumulative pregnancy rate (MD 1.73, [95% CI: 1.14, 2.60], *I^2^* = 36%, *p* = 0.009) (**Figure 3**). All the results above are listed in Table 2.





**FIGURE 3** Forest plot of studies evaluating optimal embryos (A); fertilization rate (B); live birth rate (C); cleavage rate (D); embryo implantation rate (E); E_2_ on the day of hCG (F); early miscarriage rate (G); LH on the day of hCG (H); cumulative pregnancy rate (I); duration of gonadotropin (J); total dose of gonadotropin (K).

**TABLE 2.** The summary results of forest plot for clinical outcomes.

| **Clinical outcomes** | **Studies (n)** | **Case (n)** | **OR/SMD/MD 95% CI** | *p* | ***I^2^* (%)** | **Model** |
| --- | --- | --- | --- | --- | --- | --- |
| Cycle cancellation rate | 10 | 3,132 | 0.78 [0.64, 0.95] | 0.02 | 41 | Fixed |
| Clinical pregnancy rate | 7 | 1,096 | 1.39 [1.01, 1.91] | 0.05 | 0 | Fixed |
| Number of oocytes retrieved | 9 | 2,593 | 0.30 [-0.03, 0.62] | 0.08 | 78 | Random |
| Premature LH surge rate | 4 | 1,905 | 0.10 [0.07, 0.15] | <0.00001 | 0 | Fixed |
| Optimal embryos rate | 5 | 1,517 | 1.50 [1.20, 1.88] | 0.0004 | 44 | Fixed |
| Fertilization rate | 3 | 1,722 | 1.14 [0.90, 1.43] | 0.28 | 0 | Fixed |
| Live birth rate | 3 | 513 | 1.54 [0.94, 2.51] | 0.09 | 13 | Fixed |
| Cleavage rate | 3 | 1,058 | 1.31 [0.61, 2.79] | 0.49 | 5 | Fixed |
| Embryo implantation rate | 4 | 965 | 1.06 [0.73, 1.55] | 0.76 | 0 | Fixed |
| E_2_ on the day of hCG | 7 | 1,505 | -0.11 [-0.29, 0.07] | 0.21 | 59 | Random |
| Early miscarriage rate | 6 | 279 | 0.74 [0.39, 1.40] | 0.35 | 0 | Fixed |
| LH on the day of hCG | 8 | 1,621 | -0.81 [-1.10, -0.53] | <0.00001 | 84 | Random |
| Cumulative pregnancy rate | 4 | 467 | 1.73 [1.14, 2.60] | 0.009 | 36 | Fixed |
| Duration of gonadotropin used | 8 | 2,441 | 1.56 [0.47, 2.66] | 0.005 | 97 | Random |
| Total dose of gonadotropin | 9 | 3,180 | 1.51 [0.90, 2.12] | <0.00001 | 98 | Random |

**3.5 Publication Bias**

Begg’s and Egger’s tests were applied to detect hidden publication bias. Regarding cycle cancellation rate, the form of the funnel plots with a symmetrical appearance was checked. The P value of Begg’s and Egger’s tests were 0.210 and 0.079, respectively, for cycle cancellation rate. Therefore, there was no meaningful publication bias in the meta-analysis (**Figure 2**).



**FIGURE 2** Begg’s test (A) and Egger’s test for cycle cancellation rate.

**4 Discussion**

DOR women are always likely to experience a premature LH surge because they usually possess fewer antral follicles which develop and mature rapidly and are vulnerable to premature luteinization (40). Clinically, conventional IVF protocols often provide unsatisfying results, such as disappointing embryo quality, the number of oocytes retrieved, and total embryos (42,43). Meanwhile, patients with DOR are were more difficult to obtain an expected result in managing LH surge than those with normal ovarian reserve (43). Therefore, it is essential to identify an effective ovarian stimulation protocol to better the outcome of DOR women with ART treatment. Currently, PPOS, first proposed by Kuang et al. (44) in 2015 to compare pregnancy outcomes for women undergoing IVF/ICSI with frozen embryo transfer, has drawn our interest since it is more superior to short protocol in preventing premature LH surges and as effective as short protocol in improving IVF/ICSI outcomes. The main administration of exogenous progesterone used in PPOS is dydrogesterone (DYG), progesterone capsules (PC), and medroxyprogesterone acetate (MPA) (45). In recent years, PPOS has been wildly widely recognized as a vital protocol for ovarian stimulation, especially in women with DOR (43). For example, a previous self-controlled study enrolled infertile patients with DOR proved that MPA substantially suppressed LH surge and facilitated pregnancy rate, high-grade embryos, MII oocytes, normal fertilized oocytes, and live birth rate compared to CC protocol, which may involve complex molecular mechanisms (46). Substantial evidence has implied that the PPOS protocol could influence the follicular microenvironment by regulating miR-4261 and miR-6869-5p expression in granulosa cells (47). Meanwhile, MPA may increase the ovulation rate by ameliorating the mRNA expression of GJA1 and VEGF in follicles (48). Additionally, DYG could stimulate oocyte maturation and ovulation by boosting the concentrations of acylcarnitines, lysophospholipids, urea, putrescine, and free amino acids via the purinergic signaling and arachidonic acid metabolic pathway in ovary (49). Still, the mechanism underlying progestin that ameliorates outcomes for women with DOR is not elaborated clearly. Consequently, further research is required to investigate the exact mechanism.

However, so far, there was no evidence-based medical support to inform the use of PPOS for patients with DOR undergoing IVF/ICSI. Thus, this meta-analysis was performed to explore the value of PPOS on patients with DOR. In this study, we included 14 articles involving 4182 women with DOR. According to the pooled results of the study, forest plots distinctly presented that the use of PPOS could notably reduce the incidence of cycle cancellation rate and increase clinical pregnancy rate. On the other hand, our result without heterogeneity demonstrated that there was a significant value in preventing premature LH surge with PPOS applied. Regarding the secondary outcomes, women with DOR treated with PPOS were significantly associated with superior optimal embryos rate, lower LH on the day of hCG, increase in the duration and the amount of gonadotropins required, and a higher incidence of cumulative pregnancy rate. Nonetheless, there is absence of evidence to recommend that PPOS protocol is correlated with a considerable difference in the number of oocytes retrieved, fertilization rate, live birth rate, cleavage rate, embryo implantation rate, E_2_ on the day of hCG, and early miscarriage rate compared to CC/LE plus gonadotropin protocols. In addition, according to Begg’s and Egger’s tests, no publication bias existed among the studies, and each result was also estimated by sensitivity analysis, which indicated that our results are stable robust and reliable. Taken together, we consider PPOS to be an effective protocol for patients with DOR, based on the high-quality evidence above, which might be valuable for clinicians to choose ovarian stimulation strategies.

A previous meta-analysis (43) focusing on PPOS for patients in ART demonstrated that PPOS is profitable for women with different ovarian reserve. However, it only searched four databases and included nine articles published before 2020. Among the nine studies included, solely two studies with 544 cases compared the difference of PPOS with natural cycle or antagonist protocol. Therefore, their results ought to be interpreted with caution as the different control protocols and small sample sizes were included. To our knowledge, this is the first meta-analysis to investigate the clinical value of PPOS for women with DOR undergoing IVF or ICSI compared to CC/LE plus gonadotropin stimulation. Our study has several strengths. First, this meta-analysis included only women with DOR receiving PPOS or CC/LE plus gonadotropin stimulation during IVF or ICSI; thus, this research more specifically mirrored the value of PPOS in this unique population. Second, we carefully screened nine databases and included 4182 women with DOR in this analysis. The databases enrolled were more comprehensive, and the sample size was larger than in the previous study as well. Third, the earlier meta-analysis (43) failed to check publication bias and stability of their conclusions. Instead, we used Begg’s and Egger’s tests, along with sensitivity analysis, to verify our results. Hence, we are convinced that our conclusions are more suitable for clinical practice.

Yet, several limitations should be acknowledged frankly. First of all, the 14 articles selected were retrospective studies, which may exist certain biases and, to some extent, generate a weak evidence grade compared to randomized controlled trials (RCTs). Whereas the NOS results presented that all studies included were high-quality. Secondly, all studies failed to report the adverse effects, such as ovarian hyperstimulation syndrome and deep vein thrombosis during the use of PPOS; thus, the safety of PPOS can not be estimated by meta-analysis, which might be an inherent deficiency of this study. Thirdly, significant heterogeneities could still be noticed in some outcomes, like the duration and total dose of gonadotropin used, although we performed subgroup analysis. We consider the results may be explained by the different amounts of gonadotropin applied for each patient according to the concentrations of sexual hormones and the size and quantity of developing follicles. Lastly, four studies utilized the median and 25th - 75th percentiles for continuous variables with skewed distributions, which enabled us cannot pool their related data into our analysis. Hence, more high-quality multicenter RCTs are required to confirm the value of PPOS for patients with DOR.

**5 Conclusion**

In summary, the results of this systematic review and meta-analysis confirmed that PPOS might improve clinical pregnancy rate, optimal embryos rate, and cumulative pregnancy rate for women with DOR who are undergoing IVF/ ICSI. In addition, PPOS might decrease cycle cancellation rate, LH level on the day of hCG, and premature LH surge rate for DOR patients. This would benefit clinicians in adjusting ovarian stimulation strategy. However, the duration and the amount of gonadotropins required were higher with the PPOS protocol. Therefore, we suggest that women with DOR undergoing IVF/ICSI should be appropriately evaluated before receiving the PPOS protocol.

**References**

1. Boivin J, Bunting L, Collins JA, et al. International estimates of infertility prevalence and treatment-seeking: potential need and demand for infertility medical care. *Hum Reprod.* (2007) 22(6):1506-12.

2. Inhorn MC, Patrizio P. Infertility around the globe: new thinking on gender, reproductive technologies and global movements in the 21st century. *Hum Reprod Update.* (2015) 21(4):411-26. doi: 10.1093/humupd/dmv016.

3. Carson SA, Kallen AN. Diagnosis and Management of Infertility: A Review. *JAMA.* (2021) 326(1):65-76. doi:10.1001/jama.2021.4788.

4. Devine K, Mumford SL, Wu M, et al. Diminished ovarian reserve in the United States assisted reproductive technology population: diagnostic trends among 181,536 cycles from the Society for Assisted Reproductive Technology Clinic Outcomes Reporting System. *Fertil Steril.* (2015) 104(3). doi: 10.1016/j.fertnstert.2015.05.017.

5. Jaswa EG, McCulloch CE, Simbulan R, et al. Diminished ovarian reserve is associated with reduced euploid rates via preimplantation genetic testing for aneuploidy independently from age: evidence for concomitant reduction in oocyte quality with quantity. *Fertil Steril.* (2021) 115(4):966-73. doi: 10.1016/j.fertnstert.2020.10.051.

6. Christodoulaki A, Boel A, Tang M, et al. Prospects of Germline Nuclear Transfer in Women With Diminished Ovarian Reserve. *Front Endocrinol (Lausanne).* (2021) 12:635370. doi: 10.3389/fendo.2021.635370.

7. Zhang W, Zhang L, Liu Y*, et al.* Higher chromosomal aberration frequency in products of conception from women older than 32 years old with diminished ovarian reserve undergoing IVF/ICSI. *Aging (Albany NY).* (2021) 13(7):10128-40. doi: 10.18632/aging.202772.

8. Arnanz A, Bayram A, Elkhatib I*, et al.* Antimüllerian hormone (AMH) and age as predictors of preimplantation genetic testing for aneuploidies (PGT-A) cycle outcomes and blastocyst quality on day 5 in women undergoing in vitro fertilization (IVF). *J Assist Reprod Genet.* (2023). doi: 10.1007/s10815-023-02805-z.

9. Kawwass JF, Hipp HS, Session DR, et al. Severity of Diminished Ovarian Reserve and Chance of Success with Assisted Reproductive Technology. *J Reprod Med.* (2017) 62(3-4):153-60.

10. Ferraretti AP, La Marca A, Fauser BCJM , et al. ESHRE consensus on the definition of 'poor response' to ovarian stimulation for in vitro fertilization: the Bologna criteria. *Hum Reprod.* (2011) 26(7):1616-24. doi: 10.1093/humrep/der092.

11. Singh R, Kaur S, Yadav S, et al. Gonadotropins as pharmacological agents in assisted reproductive technology and polycystic ovary syndrome. *Trends Endocrinol Metab.* (2023) 34(4):194-215. doi: 10.1016/j.tem.2023.02.002.

12. Baker VL, Brown MB, Luke B, et al. Gonadotropin dose is negatively correlated with live birth rate: analysis of more than 650,000 assisted reproductive technology cycles. *Fertil Steril.* (2015) 104(5). doi: 10.1016/j.fertnstert.2015.07.1151.

13. Moffat R, Hansali C, Schoetzau A*, et al.* Randomised controlled trial on the effect of clomiphene citrate and gonadotropin dose on ovarian response markers and IVF outcomes in poor responders. *Hum Reprod.* (2021) 36(4):987-97. doi: 10.1093/humrep/deaa336.

14. Chen Q, Chai W, Wang Y*, et al.* Progestin vs. Gonadotropin-Releasing Hormone Antagonist for the Prevention of Premature Luteinizing Hormone Surges in Poor Responders Undergoing in vitro Fertilization Treatment: A Randomized Controlled Trial. *Front Endocrinol (Lausanne).* (2019) 10:796. doi: 10.3389/fendo.2019.00796.

15. Xu B, Wang J, Xia L, et al. Increased Uterine NK cell numbers and perforin expression during the implantation phase in IVF Cycles with GnRH Antagonist Protocol. *Sci Rep.* (2017)7:39912. doi: 10.1038/srep39912.

16. Kato K, Ezoe K, Yabuuchi A, et al. Comparison of pregnancy outcomes following fresh and electively frozen single blastocyst transfer in natural cycle and clomiphene-stimulated IVF cycles. *Hum Reprod Open*. (2018) 2018(3):hoy006. doi:10.1093/hropen/hoy006

17. Liu R, Zhou L, Chen X, et al. Letrozole Supplementation and the Increased Risk of Elevated Progesterone Levels on Trigger Day. *Front Endocrinol (Lausanne)*. (2022) 13:904089. doi:10.3389/fendo.2022.904089

18. La Marca A, Capuzzo M, Sacchi S, *et al.* Comparison of euploidy rates of blastocysts in women treated with progestins or GnRH antagonist to prevent the luteinizing hormone surge during ovarian stimulation. *Hum Reprod.* (2020) 35(6):1325-31. doi: 10.1093/humrep/deaa068.

19. Kalafat E, Turkgeldi E, Yıldız S, et al. Outcomes of a GnRH Agonist Trigger Following a GnRH Antagonist or Flexible Progestin-Primed Ovarian Stimulation Cycle. *Front Endocrinol (Lausanne).* (2022) 13:837880. doi: 10.3389/fendo.2022.837880.

20. Zhou R, Dong M, Huang L*, et al.* Comparison of cumulative live birth rates between progestin-primed ovarian stimulation protocol and gonadotropin-releasing hormone antagonist protocol in different populations. *Front Endocrinol (Lausanne).* (2023) 14:1117513. doi: 10.3389/fendo.2023.1117513.

21. Dong J, Wang Y, Chai WR*, et al.* The pregnancy outcome of progestin-primed ovarian stimulation using 4 versus 10 mg of medroxyprogesterone acetate per day in infertile women undergoing in vitro fertilisation: a randomised controlled trial. *BJOG.* (2017) 124(7):1048-55. doi: 10.1111/1471-0528.14622. Huang J, Xie Q, Lin J, *et al*. Neonatal outcomes and congenital malformations in children born after dydrogesterone application in progestin-primed ovarian stimulation protocol for IVF: a retrospective cohort study. *Drug Des Devel Ther*. (2019) 26;13:2553-2563. doi: 10.2147/DDDT.S210228.

22. Liu Y, Nan Y, Chen F, et al. Application effect of different ovarian stimulation protocols in elderly patients with diminished ovarian reserve. *Journal of Xinxiang Medical University.* (2020) 37(5):465-70. doi: 10.7683/xxyxyxb.2020.05.016.

23. Zhao Hj, Zhang Xh, Chen J, et al. Comparison of Three Controlled Ovarian Hyperstimulation Protocols in Advanced-Age Infertile Patients with Diminished Ovarian Reserve. *Journal of International Reproductive Health/Family Planning.* (2023) 42(1):13-7. doi: 10.12280/gjszjk.20220213.

24. Fu Yh, Yang Tc, Xi Ht, et al. Effect of progestin-primed ovarian stimulation scheme in patients with diminished ovarian reserve. *Journal of Wenzhou Medical University.* (2017) 47(4):258-62. doi: 10.3969/j.issn.2095-9400.2017.04.006.

25. Zhang C, Zhu P, Yan Wq, et al. Progestinprimed ovarian stimulation protocol in women with poor ovarian reserve undergone IVF/ICSI programmes. *Chinese Journal of Birth Health & Heredity.* (2016) 24(10):111-4.

26. Page MJ, McKenzie JE, Bossuyt PM, *et al.* The PRISMA 2020 statement: an updated guideline for reporting systematic reviews. *BMJ.* (2021) 372:n71. doi: 10.1136/bmj.n71.

27. Cohen J, Chabbert-Buffet N, Darai E. Diminished ovarian reserve, premature ovarian failure, poor ovarian responder--a plea for universal definitions. *J Assist Reprod Genet.* (2015) 32(12):1709-12. doi: 10.1007/s10815-015-0595-y.

28. Consensus on clinical diagnosis and management of diminished ovarian reserve. *Journal of Reproductive Medicine.* (2022) 31(4):425-34.

29. Stang A. Critical evaluation of the Newcastle-Ottawa scale for the assessment of the quality of nonrandomized studies in meta-analyses. *Eur J Epidemiol.* (2010) 25(9):603-5. doi: 10.1007/s10654-010-9491-z.

30. Tu X, You B, Jing M, et al. Progestin-Primed Ovarian Stimulation Versus Mild Stimulation Protocol in Advanced Age Women With Diminished Ovarian Reserve Undergoing Their First In Vitro Fertilization Cycle: A Retrospective Cohort Study. *Front Endocrinol (Lausanne).* (2021) 12:801026. doi: 10.3389/fendo.2021.801026.

31. Zhao Ss, Peng Z, Tu Zy, et al. Clinical analysis of IVF/ICSI-ET assisted pregnancy therapy in elder patients with diminished ovarian reserve. *Journal of Reproductive Medicine.* (2023) 32(3):350-6. doi: 10.3969/j.issn.1004-3845.2023.03.008.

32. Yu Cm, Dai Xl, Wang Yf, et al. Effect of PPOS protocol on IVF-ET outcomes in different ages patients with DOR. *Maternal & Child Health Care of China.* (2019) 34(17):4043-5. doi: 10.7620/zgfybj.j.issn.1001-4411.2019.17.53.

33. Fan Xy, Yu S, Wang Y, et al. Controllability comparison between progestin-primed ovarian stimulation and mild stimulation protocols in patients with diminished ovarian reserve in oocytes retrieval cycle. *Chinese Journal of Clinical Medicine.* (2021) 28(4):539-43. doi: 10.12025/j.issn.1008-6358.2021.20210409.

34. Yang Zx, Wen Y, Su Cm, *et al.* Comparison the Clinical Outcomes of Progestin-Primed Ovarian Stimulation during Follicular Period and Mild-stimulation in Patients with Diminished Ovarian Reserve. *Journal of Kunming Medical University.* (2022) 43(12):53-7. doi: 10.12259/j.issn.2095-610X.S20221211.

35. Zeng Ql, Yan L, Li Yp, et al. Comparison of clinical outcomes and economic benefits between progestin-primed ovarian stimulation and micro-stimulation in patients with diminished ovarian reserve. *Chinese Journal of Clinical Research.* (2020) 33(7):908-12. doi: 10.13429/j.cnki.cjcr.2020.07.010.

36. Zheng J, Zhou Lm, sun Yt, et al. Comparison of the clinical outcomes of three ovulation induction protocols in elderly patients with decline in ovarian reserve. *Chinese Journal of Reproduction and Contraception.* (2020) 40(3):194-200. doi: 10.3760/cma.j.cn101441-20190325-00121.

37. Wang Cl, Xu J. Clinical efficacy of modified naturalcycle for treating women with ovarian reserve dysfunction. *Chinese Journal of Family Planning.* (2020) 28(11):1864-7. doi: 10.3969/j.issn.1004-8189.2020.11.033.

38. Xu Xy. Analysis of clinical outcomes of different ovulation stimulation protocols in elderly patients with diminished ovarian reserve. *Zhengzhou University.* (2021).

39. Yu G, Xiang Yg, Li Y, et al. Application of progestin-primed ovarian stimulation or mild stimulation protocols in patients with diminished ovarian reserve. *Chinese Journal of Practical Gynecology and Obstetrics.* (2017) 33(5):502-6. doi: 10.19538/j.fk2017050116.

40. Wu Y-G, Barad DH, Kushnir VA, *et al.* Aging-related premature luteinization of granulosa cells is avoided by early oocyte retrieval. *J Endocrinol.* (2015) 226(3):167-80. doi: 10.1530/JOE-15-0246.

41. Datta AK, Maheshwari A, Felix N, et al. Mild versus conventional ovarian stimulation for IVF in poor, normal and hyper-responders: a systematic review and meta-analysis. *Hum Reprod Update.* (2021) 27(2):229-53. doi: 10.1093/humupd/dmaa035.

42. Mizrachi Y, Horowitz E, Farhi J, et al. Ovarian stimulation for freeze-all IVF cycles: a systematic review. *Hum Reprod Update.* (2020) 26(1):118-35. doi: 10.1093/humupd/dmz037.

43. Guan S, Feng Y, Huang Y, et al. Progestin-Primed Ovarian Stimulation Protocol for Patients in Assisted Reproductive Technology: A Meta-Analysis of Randomized Controlled Trials. *Front Endocrinol (Lausanne).* (2021) 12:702558. doi: 10.3389/fendo.2021.702558.

44. Kuang Y, Chen Q, Fu Y*, et al.* Medroxyprogesterone acetate is an effective oral alternative for preventing premature luteinizing hormone surges in women undergoing controlled ovarian hyperstimulation for in vitro fertilization. *Fertil Steril.* (2015) 104(1). doi: 10.1016/j.fertnstert.2015.03.022.

45. Alexandru P, Cekic SG, Yildiz S, et al. Progestins versus GnRH analogues for pituitary suppression during ovarian stimulation for assisted reproductive technology: a systematic review and meta-analysis. *Reprod Biomed Online.* (2020) 40(6):894-903. doi: 10.1016/j.rbmo.2020.01.027.

46. Yu C-M, Dai X-L, Wang Y-F, *et al.* Progestin-primed ovarian stimulation improves the outcomes of IVF/ICSI cycles in infertile women with diminished ovarian reserve. *J Chin Med Assoc.* (2019) 82(11):845-8. doi: 10.1097/JCMA.0000000000000177.

47. Yu J, Zhu D, Zeng C, et al. MicroRNA expression profiles in the granulosa cells of infertile patients undergoing progestin primed ovarian stimulation. *Eur J Obstet Gynecol Reprod Biol.* (2022) 276:228-35. doi: 10.1016/j.ejogrb.2022.08.001.

48. Seekallu SV, Toosi BM, Grazul-Bilska AT, et al. Markers of ovarian antral follicular development in sheep: comparison of follicles destined to ovulate from the final or penultimate follicular wave of the estrous cycle. *Reproduction.* (2010) 140(4):559-68. doi: 10.1530/REP-10-0064.

49. Jiang Y-X, Shi W-J, Ma D-D, *et al.* Dydrogesterone exposure induces zebrafish ovulation but leads to oocytes over-ripening: An integrated histological and metabolomics study. *Environ Int.* (2019) 128:390-8. doi: 10.1016/j.envint.2019.04.059.
